# Transitional Cell Carcinoma in Orthotopic Ileal Neobladder

**DOI:** 10.1155/2014/218615

**Published:** 2014-11-18

**Authors:** Ozgur Cakmak, Huseyin Tarhan, Orcun Celik, Ulku Kucuk, Yusuf Ozlem Ilbey

**Affiliations:** ^1^Urology Department, Tepecik Training and Research Hospital, 35540 Izmir, Turkey; ^2^Pathology Department, Tepecik Training and Research Hospital, 35540 Izmir, Turkey

## Abstract

Urothelial carcinoma developing in orthotopic ileal neobladder is an extremely rare entity. Fewer than 10 cases have been reported in the literature describing urothelial carcinoma recurrence in orthotopic ileal neobladder. We report a case of transitional cell carcinoma recurrence in orthotopic ileal neobladder after 11 years of surgery.

## 1. Introduction

Secondary malignancies especially adenocarcinomas following some type of urinary diversions using isolated gut segments are not an uncommon phenomenon. Several reports have been published regarding tumor growth in urinary diversions as well as conduits, cystoplasties, continent reservoirs, and ileal neobladders. Although some cases of urothelial carcinoma developing in colonic diversions have been published, urothelial carcinoma developing in an orthotopic ileal neobladder has been rarely reported [1]. We present a patient with transitional cell carcinoma (TCC) developing in an ileal neobladder after 11 years of radical cystoprostatectomy and W-shaped ileal neobladder reconstruction.

## 2. Case Report

**Figure 1:**
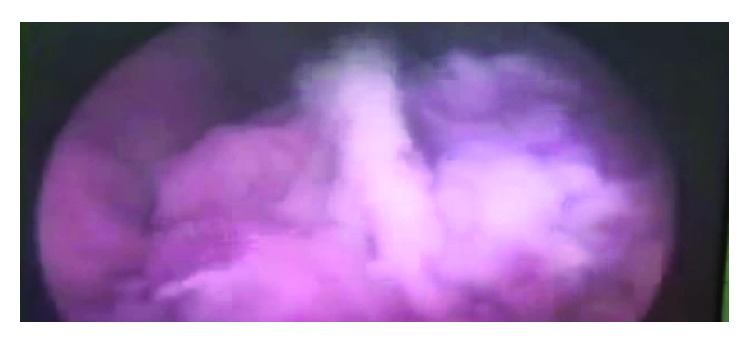
Cystoscopic view of lesions in the neobladder depicting tumors with papillary configuration.

In May 2002 a 40-year-old man had been diagnosed with grade 2 urothelial carcinoma of the bladder with lamina propria invasion (pT1) after investigations for painless macroscopic hematuria of 4 months' duration. Three recurrences within four years occurred. While first two transurethral resection (TUR) revealed grade 2, pT1 disease, last TUR indicated grade 3 pT1 bladder cancer. Radical cystoprostatectomy and W-shaped ileal neobladder reconstruction was performed due to extensive and multiple papillary tumor in the bladder. No cancer was detected in prostatic urethra and all surgical margins were negative (grade 3, stage pT1N0M0). Patient reported gross hematuria after 11 years of uneventful follow-up period. Cystoscopic examination revealed multiple papillary tumors localised in the neobladder especially in the region of the base and left lateral side of the pouch. No obvious pathology was seen in urethra and urethral-neobladder anastomosis (Figure 1). His laboratory findings and abdominal computed tomography did not indicate any pathology.

**Figure 2:**
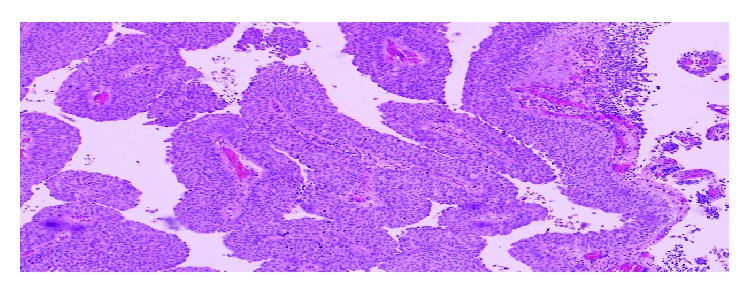
Biopsy from the tumoral areas of the neobladder: papillary tumoural growth with low grade nuclear features (H&E ×200).

**Figure 3:**
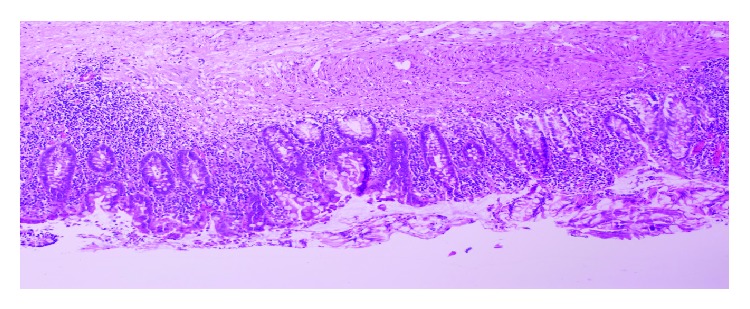
Biopsy from the areas of the nontumoral neobladder: small intestinal mucosa showing villous atrophy and inflammation in the lamina propria (H&E ×200).

Transurethral biopsy of the tumor, urethra, urethral-neobladder anastomosis, and colonic mucosa was performed. Histopathologic examination of the lesion revealed low grade transitional cell carcinoma (Figure 2). No tumor was reported in the urethra, urethral-neobladder anastomosis, and colonic mucosa (Figure 3). We offered resection of neobladder with creation of an ileal conduit which was rejected by the patient. TUR of the lesion was completed. Histopathologic study of tumor in the neobladder proved low grade TCC. We followed up patient with cystoscopy and cytologic evaluation performed four times in a year and upper urinary tract imaging performed yearly. The follow-up scheme was determined empirically by us due to the lack of any guidelines about this rare condition. No tumor recurrence in the neobladder or any pathology in the upper urinary tract was detected in the one-and-half-year follow-up period.

## 3. Discussion

In this study we presented a case with TCC recurrence in an ileal neobladder after 11 years of radical cystoprostatectomy. Although there are many reports regarding seconder malignancy occurrence after ureterosigmoidostomies, cystoplasties, and intestinal conduits only few cases indicating tumor recurrence after orthotopic neobladders are present.

Different than tumors secondary to ureterosigmoidostomies which almost all are constituted from adenomas and adenocarcinomas, only 72.6% of tumors secondary to urinary diversions using isolated gut segments are adenomas and adenocarcinomas while 27.4% comprised transitional cell carcinoma, squamous cell carcinoma, signet ring cell carcinoma, small cell carcinoma, and leiomyosarcoma [2].

The risk of TCC recurrence along the entire urothelial tract after radical cystectomy is well-known entity. Urethra, urethral-neobladder anastomosis, ureterointestinal anastomosis, and upper urinary tract bear risk of secondary tumor recurrence after orthotopic neobladder substitution.

Urethra is the most frequent recurrence localisation after radical cystectomy with orthotopic neobladder substitution. The incidence of urethral recurrence after radical cystectomy for invasive transitional cell carcinoma has been reported between 0.7% and 18% in large series [3]. In our case no tumor was seen in urethra and urethral-neobladder anastomosis site.

Direct invasion and implantation are the two postulated hypotheses enlightening TCC occurrence in a bowel segment. Colonic and ileal mucosa are both susceptible to transitional cell carcinoma implantation as urothelium of the bladder; therefore, intraluminal tumor cell seeding can be considered as one of the potential factors that contribute to multifocal recurrence of TCC in the tissues other than urothelium [4].

In their study aiming to explore the underlying mechanism of urothelial carcinoma regrowth in unusual anatomic locations Herawi and colleagues reported two cases with urothelial carcinoma recurrence in the peritoneum and on the colonic mucosal surface of a neobladder. It is claimed that the droplets of shed tubular cells (seeds) apparently find bladder or nonurothelial surfaces (soil) compatible for their implantation and growth in according to “seed and soil hypothesis” which was postulated by the authors [5]. Our case also highlights the potential of transitional cell carcinoma to implant, not only in the urothelium of the bladder, but also in the ileal mucosa of a neobladder.

To our knowledge there are five reports in the literature indicating TCC recurrence after radical cystectomy with orthotopic ileal neobladder substitution in six different patients [4, 6–9].

Ide et al. presented a case with carcinoma in situ (CIS) developing in an ileal neobladder. The authors also had to perform left nephroureterectomy and total urethrectomy due to tumor recurrence in the right ureter and ureteral-neobladder anastomosis [7]. No tumor was seen in upper urinary tracts, ureters, and ureteral-neobladder anastomosis in our case.

The lack of the completeness of treatment due to the rejection of the surgery by the patient can be considered as the limitation of our report.

## 4. Conclusion

Urinary diversions using isolated gut segments bear risk of malignancy. In long-term follow-up not only adenomas and adenocarcinomas but also transitional cell carcinomas may develop in the neobladder. TCC recurrence in the neobladder should be considered in patients with haematuria who underwent radical cystectomy and orthotopic ileal neobladder.
